# Letter from L. G. Hardman, M. D.

**Published:** 1890-05

**Authors:** L. G. Hardman

**Affiliations:** Philadelphia


UNIVERSITY OF PENNSYLVANIA MEDICAL DEPARTMENT,
PHILADELPHIA, March 26, 1890.

DEAR EDITOR—I notice through the circular press here that pneumonia is very fatal, and the plan of treatment now is not as successful as is the depletion plan of many years ago, and in the article it stated, or quoted from Dr. H. C. Wood as thinking that blood letting would be of great benefit in many cases. I was at one of Dr. Jesse Tyson's clinics to-day, in which he spoke of the use of strychnia in 1-60 to 1-20 of a grain as a respiratory stimulant, and that they use it in most of the cases of pneumonia. I wish to state, in this connection, that if respiratory stimuli are beneficial in pneumonia, pleurisy and cardiac troubles, that we have in tobacco infusions, or nicotine, the best agent which belongs to that class, and so far as I know has not been spoken of before. I have given hypodermics of infusion of tobacco in the dog and it will produce panting almost immediately, and there were no injurious effects followed. I have also used it in the vein without injurious effect. I want also to mention that it is certainly a most marked physiological antagonist to opium, and would be especially useful in opium poisoning. I will give you the experiment on the dog in full soon, if so desired. Respectfully,

L. G. HARDMAN.

